# Glyceraldehyde-3-phosphate Dehydrogenase (GAPDH) Aggregation Causes Mitochondrial Dysfunction during Oxidative Stress-induced Cell Death*[Fn FN2]

**DOI:** 10.1074/jbc.M116.759084

**Published:** 2017-02-06

**Authors:** Hidemitsu Nakajima, Masanori Itakura, Takeya Kubo, Akihiro Kaneshige, Naoki Harada, Takeshi Izawa, Yasu-Taka Azuma, Mitsuru Kuwamura, Ryouichi Yamaji, Tadayoshi Takeuchi

**Affiliations:** From the ‡ Laboratory of Veterinary Pharmacology,; the § Division of Applied Life Sciences, and; the ¶ Laboratory of Veterinary Pathology, Graduate School of Life and Environmental Science, Osaka Prefecture University, Izumisano, Osaka 5988531, Japan

**Keywords:** amyloid, cell death, mitochondria, nitric oxide, protein aggregation

## Abstract

Glycolytic glyceraldehyde-3-phosphate dehydrogenase (GAPDH) is a multifunctional protein that also mediates cell death under oxidative stress. We reported previously that the active-site cysteine (Cys-152) of GAPDH plays an essential role in oxidative stress-induced aggregation of GAPDH associated with cell death, and a C152A-GAPDH mutant rescues nitric oxide (NO)-induced cell death by interfering with the aggregation of wild type (WT)-GAPDH. However, the detailed mechanism underlying GAPDH aggregate-induced cell death remains elusive. Here we report that NO-induced GAPDH aggregation specifically causes mitochondrial dysfunction. First, we observed a correlation between NO-induced GAPDH aggregation and mitochondrial dysfunction, when GAPDH aggregation occurred at mitochondria in SH-SY5Y cells. In isolated mitochondria, aggregates of WT-GAPDH directly induced mitochondrial swelling and depolarization, whereas mixtures containing aggregates of C152A-GAPDH reduced mitochondrial dysfunction. Additionally, treatment with cyclosporin A improved WT-GAPDH aggregate-induced swelling and depolarization. In doxycycline-inducible SH-SY5Y cells, overexpression of WT-GAPDH augmented NO-induced mitochondrial dysfunction and increased mitochondrial GAPDH aggregation, whereas induced overexpression of C152A-GAPDH significantly suppressed mitochondrial impairment. Further, NO-induced cytochrome *c* release into the cytosol and nuclear translocation of apoptosis-inducing factor from mitochondria were both augmented in cells overexpressing WT-GAPDH but ameliorated in C152A-GAPDH-overexpressing cells. Interestingly, GAPDH aggregates induced necrotic cell death via a permeability transition pore (PTP) opening. The expression of either WT- or C152A-GAPDH did not affect other cell death pathways associated with protein aggregation, such as proteasome inhibition, gene expression induced by endoplasmic reticulum stress, or autophagy. Collectively, these results suggest that NO-induced GAPDH aggregation specifically induces mitochondrial dysfunction via PTP opening, leading to cell death.

## Introduction

Glyceraldehyde-3-phosphate dehydrogenase (GAPDH) is a glycolytic enzyme that is responsible for the sixth step of glycolysis (1). In addition to this metabolic function, GAPDH is now recognized as a multifunctional protein that exhibits other functions, including transcriptional (2) and posttranscriptional gene regulation (3), intracellular membrane trafficking (4), and cell death (5, 6). In the GAPDH-mediated cell death pathway, the involvement of GAPDH in nuclear translocation and its aggregation under oxidative stress have been proposed (7–9). The active-site cysteine (Cys-152) seems to play a crucial role in both pathways. For example, GAPDH binds to Siah (seven *in absentia* homolog) through oxidation/*S*-nitrosylation of Cys-152 and translocates into the nucleus in response to oxidative stress, such as that from nitric oxide (NO) (10). Nuclear GAPDH activates p300/CREB (cAMP-response element-binding protein)-binding protein (CBP) (11) and poly(ADP-ribose) polymerase-1 (12). Additionally, oxidative stressors initiate amyloid-like GAPDH aggregation via intermolecular disulfide bonds at Cys-152 (13–15).

The accumulation of unfolded proteins can cause protein aggregation in the aged brain, and these aggregates facilitate the formation of pathological amyloid deposits, which is a key cause of several neurodegenerative/neuropsychiatric disorders (16, 17). Aggregated GAPDH in the brain is also amyloidogenic, and GAPDH amyloidal aggregates colocalize with Lewy bodies in Parkinson's disease (18, 19) and with senile plaques and neurofibrillary tangles in Alzheimer's disease (20–23). Based on these findings, we suggested previously a critical role for GAPDH aggregation in oxidative stress-induced neuronal cell death both *in vitro* and *in vivo* (14, 15, 24, 25). Further, GAPDH aggregation is likely related to the pathogeneses of amyotrophic lateral sclerosis and Huntington's disease (26, 27). However, the detailed mechanisms for cell death induced by GAPDH aggregation in the context of these pathogeneses remain unclear.

It has been posited that abnormal protein aggregation leads to mitochondrial dysfunction, proteasome inhibition, endoplasmic reticulum (ER)^3^ stress, and autophagy, which ultimately cause cell death (28–32). Notably, ∼5–20% of the total GAPDH under physiological conditions is generally bound to the mitochondria in most species (33, 34). Further, treatment of isolated mitochondria with GAPDH directly causes their dysfunction (35) through the activation of voltage-dependent anion channels, which are known components of the mitochondrial permeability transition pore (PTP) (36). PTP opening leads to mitochondrial depolarization and the release of cell death mediators from the intermembrane space, such as cytochrome *c* (cyt *c*) and apoptosis-inducing factor (AIF) (37). From these observations, we focused on mitochondria to elucidate the cell death pathway that is mediated by GAPDH aggregation.

We demonstrated previously that NO-induced cell death is attenuated by a GAPDH mutant with a substitution of Cys-152 to alanine (C152A-GAPDH) in a dominant-negative manner (24). Therefore, this study was designed to investigate the underlying mechanism by which this occurs. Our experiments using C152A-GAPDH revealed the involvement of mitochondrial dysfunction, as well as the subsequent release of cyt *c* and nuclear translocation of AIF via PTP opening, in NO-induced necrotic cell death mediated by GAPDH aggregation.

## Results

### 

#### 

##### Relation between NO-induced GAPDH Aggregation and Mitochondrial Dysfunction in SH-SY5Y Cells

**FIGURE 1.**
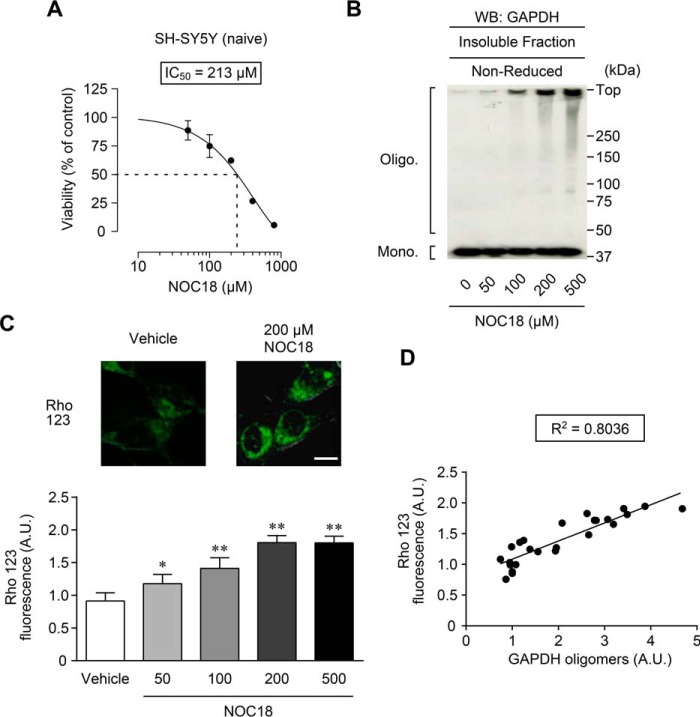
**Relation between NO-induced GAPDH aggregation and mitochondrial dysfunction in SH-SY5Y cells.** *A*, effect of treatment with the indicated concentrations of NOC18 for 48 h on cell viability in SH-SY5Y cells. Data are presented as mean ± S.D. (*n* = 3–5). *B*, concentration-dependent effect of NOC18 treatment in SH-SY5Y cells on the formation of insoluble GAPDH oligomers. Cells were treated with the indicated concentrations of NOC18 for 48 h followed by insoluble fractionation and subsequent non-reduced Western blotting (*WB*). *C*, concentration-dependent effect of NOC18 treatment on mitochondrial depolarization measured by Rho 123 fluorescence. Fluorescence images of cells treated with vehicle or 200 μm NOC18 are shown in the *top panels*. The *graph* represents Rho 123 fluorescence of cells treated with the indicated concentrations of NOC18. Values were normalized by the number of cells stained with Hoechst 33342. Data are presented as mean ± S.D. (*n* = 6). *, *p* < 0.05; **, *p* < 0.01, relative to vehicle treatment, Dunnett's test. *Scale bar*, 10 μm. *D*, correlation between the amount of GAPDH oligomers and Rho 123 fluorescence (*R*^2^ = 0.8036).

As an oxidant, we selected NOC18, an NO generator (14). The IC_50_ for NOC18-induced decrease of cell viability in SH-SY5Y cells was ∼200 μm (Fig. 1*A*). Treatment with NOC18 caused GAPDH oligomer formation in cells and in insoluble fractions in a concentration-dependent manner (Fig. 1*B*). To validate the effect of GAPDH aggregation on mitochondria, we assessed mitochondrial membrane potential in cells using the anion dye rhodamine 123 (Rho 123). Compared with vehicle treatment, 200 μm NOC18 significantly increased Rho 123 fluorescence, indicating a disruption of the mitochondrial membrane, which was enhanced in a concentration-dependent manner with NOC18 (Fig. 1*C*). We next examined the correlation between the formation of GAPDH oligomers and the disruption of mitochondrial membrane potential; the result clearly indicated a strong correlation (*R*^2^ = 0.8036, Fig. 1*D*). These results indicate a relation between NO-induced GAPDH aggregation and mitochondrial dysfunction. Thus, in the following experiments, sufficient aggregates of GAPDH and mitochondrial dysfunction were observed after 48 h of treatment with 200 μm NOC18.

##### Formation of GAPDH Aggregates Occurs at Mitochondria

**FIGURE 2.**
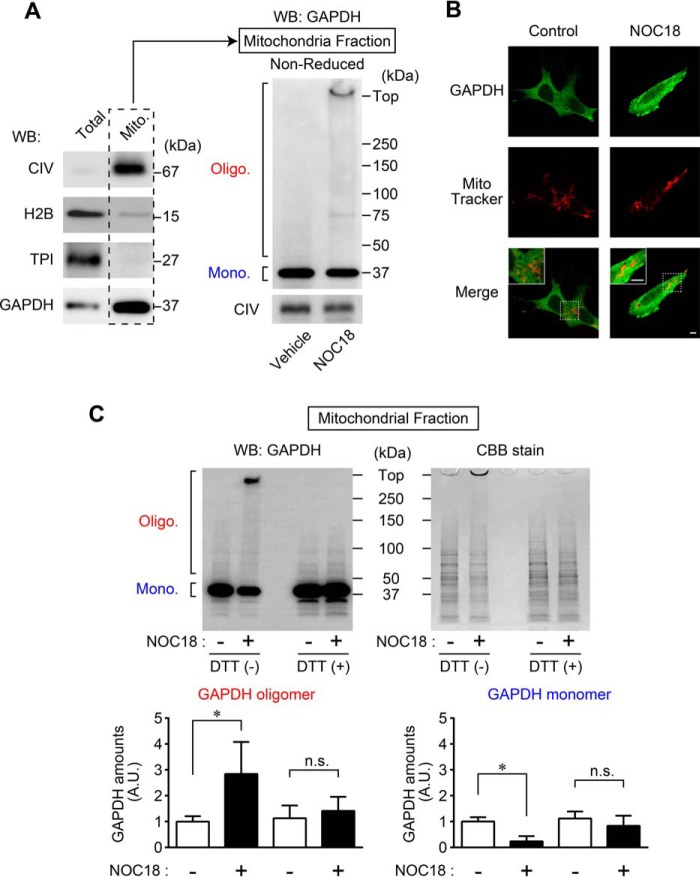
**Formation of GAPDH aggregates occurs at mitochondria.** *A*, confirmation of mitochondrial fractionation using CIV as a mitochondrial marker, H2B as a nuclear marker, and TPI as a cytosolic marker. GAPDH was present in the mitochondrial fractions (*left panel*) from SH-SY5Y cells, which were subjected to non-reduced Western blotting (*WB*) for GAPDH (*right panel*). *B*, images of cell immunofluorescence of GAPDH (*green*) and MitoTracker Red (*red*) are shown. GAPDH-positive punctate signals in cells treated with NOC18 partially colocalize with MitoTracker Red. *Scale bar*, 5 μm. *C*, Western blotting for GAPDH and Coomassie Brilliant Blue (CBB) staining of mitochondrial fractions in the absence (non-reduced) or presence (reduced) of 100 mm DTT. The *graphs* represent quantitative results of Western blotting analysis regarding GAPDH oligomers (*Oligo*.) and monomers (*Mono*.). Data are mean ± S.D. (*n* = 3). *, *p* < 0.05, relative to NOC18(−), Student's *t* test.

To investigate the origin of the aggregates of GAPDH that induce mitochondrial dysfunction, we used Western blotting to study whether these aggregates exist within mitochondrial fractions in NOC18-treated SH-SY5Y cells (Fig. 2*A*). Successful mitochondrial fractionation was confirmed by the presence of cytochrome *c* oxidase (complex IV (CIV)) and the absence of histone H2B (a marker for nuclear fraction) and triosephosphate isomerase (a marker for cytosolic fraction). A large amount of GAPDH was present in the mitochondrial fraction, as reported previously (Fig. 2*A*, *left panel*) (35). Further, we verified the presence of GAPDH aggregates in the mitochondrial fraction from SH-SY5Y cells. Although vehicle-treated control cells had only monomeric GAPDH (37 kDa), GAPDH oligomers and a lesser number of GAPDH monomers were present in the mitochondrial fraction of NOC18-treated cells (Fig. 2*A*, *right panel*). Similarly, we used immunofluorescence to assess whether GAPDH was localized near the mitochondria using the cell-permeable mitochondrial-selective dye MitoTracker Red (Fig. 2*B*). Whereas the juxtaposition of the GAPDH signal was around the mitochondria in vehicle-treated cells, NOC18-treated cells showed punctate GAPDH-positive signals that were consistent with aggregates of GAPDH, which partially merged with MitoTracker Red, indicating mitochondrial localization of GAPDH aggregates (Fig. 2*B*, *right panels*). To identify where the aggregates of GAPDH occurred in cells, we examined the amounts of oligomeric GAPDH in mitochondrial fractions with or without DTT, which reduces GAPDH oligomers and causes dissociation to monomers (15). In the absence of DTT (under a non-reduced condition), treatment with NOC18 significantly increased the amount of GAPDH oligomers and decreased the amount of GAPDH monomers in mitochondrial fractions, whereas in the presence of DTT (under a reduced condition), these changes were abolished, as assessed by both Western blotting and Coomassie Brilliant Blue staining (Fig. 2*C*). These results suggest that NO-induced aggregates are derived from mitochondrial GAPDH.

##### NO-induced GAPDH Aggregation Directly Causes Mitochondrial Dysfunction in Vitro

**FIGURE 3.**
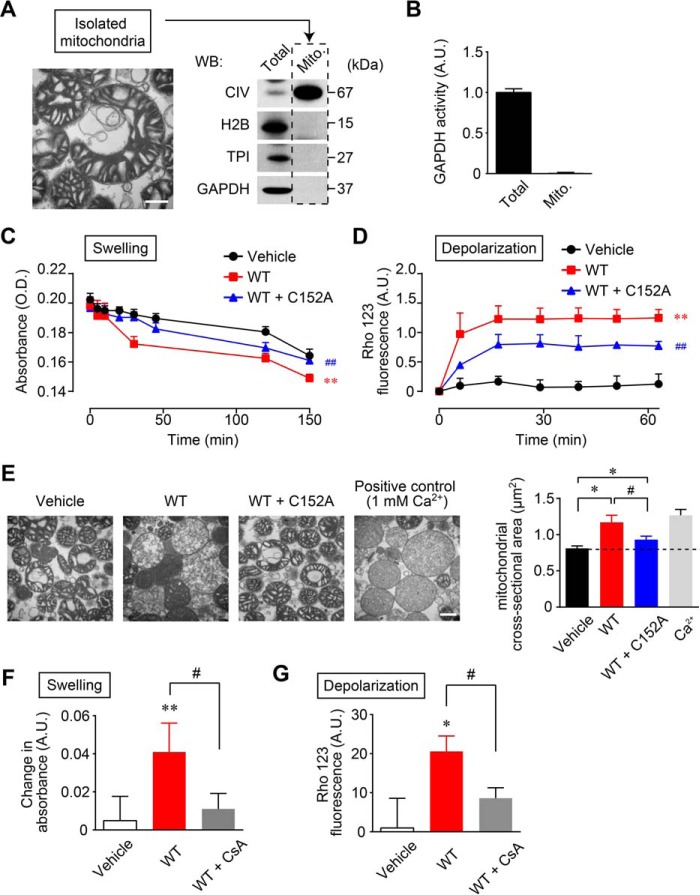
**NO-induced aggregates of GAPDH cause mitochondrial dysfunction *in vitro*.** *A*, transmission electron microscopy of isolated mitochondria (*left panel*). *Scale bar*, 400 nm. Western blotting (*WB*) shows the absence of GAPDH in isolated mitochondria (*right panel*). *B*, measurement of GAPDH enzyme activity confirms the absence of GAPDH in isolated mitochondria. *C* and *D*, effects of GAPDH aggregation on mitochondrial swelling (*C*) and depolarization (*D*). Isolated mitochondria were treated with vehicle (*black line* and *circles*), aggregates of WT-GAPDH (0.3 mg/ml, *red line* and *squares*), or an aggregate mixture of WT- (0.3 mg/ml) and C152A-GAPDH (0.75 mg/ml, *blue line* and *triangles*) for the indicated time periods. Mitochondrial swelling was measured by absorbance at 540 nm. Mitochondrial depolarization was measured by Rho 123 fluorescence. Data are mean ± S.D. (*n* = 4). **, *p* < 0.01, relative to vehicle treatment; ##, *p* < 0.01, relative to treatment with aggregates of WT-GAPDH, Dunnett's test. *E*, transmission electron microscopy of isolated mitochondria treated with vehicle, aggregates of WT-GAPDH, aggregates derived from a mixture of WT- and C152A-GAPDH, and 1 mm Ca^2+^ (*left panels*). *Scale bar*, 400 nm. A mitochondrial cross-sectional area was determined (*right*). Data are mean ± S.D. (*n* = 4). *, *p* < 0.05, relative to treatment with vehicle; #, *p* < 0.05, relative to treatment with aggregates of GAPDH, Student's *t* test. *F* and *G*, effect of CsA on GAPDH aggregate-induced mitochondrial swelling and depolarization. Isolated mitochondria were treated with vehicle or aggregates of GAPDH (0.3 mg/ml) or CsA (1 μm) for 2 min prior to treatment with aggregates of GAPDH and incubated for 30 min. Mitochondrial swelling and depolarization were measured as described for *C* and *D* above. Data are mean ± S.D. (*n* = 4). *, *p* < 0.05, and **, *p* < 0.01, relative to the treatment with vehicle, Dunnett's test; #, *p* < 0.05, relative to the treatment with aggregates of GAPDH, Student's *t* test.

We next evaluated whether GAPDH aggregation leads directly to mitochondrial dysfunction. It has been reported that the detectable amount of GAPDH bound to mitochondria differs depending on the method of isolation (34). Therefore, we attempted to obtain GAPDH-free mitochondria to accurately assess the direct action of GAPDH aggregates on mitochondria. According to the protocol reported previously (38), successful isolation of mitochondrial fractions was achieved and confirmed by transmission electron microscopy (Fig. 3*A*, *left panel*). The absence of GAPDH in the isolated mitochondria was ascertained by Western blotting (Fig. 3*A*, *right panel*) and by measuring GAPDH enzyme activity (Fig. 3*B*). We showed recently that the addition of the GAPDH mutant C152A-GAPDH inhibits NO-induced amyloidogenic aggregation of wild type GAPDH (WT-GAPDH) *in vitro* (24). Therefore, we treated the solutions of isolated mitochondria with aggregates of WT-GAPDH or a mixture containing aggregates of WT- and C152A-GAPDH. Mitochondrial dysfunction was monitored by the degree of mitochondrial swelling and mitochondrial membrane depolarization. The treatment of isolated mitochondria with aggregates of WT-GAPDH significantly decreased the turbidity of the solutions, indicating mitochondrial swelling (Fig. 3*C*, *red line* and *squares*) relative to vehicle treatment (Fig. 3*C*, *black line* and *circles*). The mixture containing aggregates of WT- and C152A-GAPDH significantly reduced the degree of swelling compared with that of WT-GAPDH alone (Fig. 3*C*, *blue line* and *triangles*). We also found that treatment with aggregates of WT-GAPDH caused robust mitochondrial membrane depolarization, indicated by an increase in Rho 123 fluorescence (Fig. 3*D*, *red line* and *squares*), and the mixture containing aggregates of WT- and C152A-GAPDH significantly attenuated this mitochondrial membrane depolarization (Fig. 3*D*, *blue line* and *triangles*). Further, we morphologically examined the effects of aggregates of GAPDH on isolated mitochondria using transmission electron microscopy (Fig. 3*E*, *left panels*), and mitochondrial swelling was assessed quantitatively (Fig. 3*E*, *right graph*). Compared with vehicle treatment, marked mitochondrial swelling was observed after treatment with aggregates of WT-GAPDH, whereas treatment with a mixture containing aggregates of WT- and C152A-GAPDH attenuated but did not abolish the formation of this abnormal morphology (Fig. 3*E*). Similar to treatment with aggregates of WT-GAPDH, the swelling of isolated mitochondria was observed after treatment with 1 mm Ca^2+^, which was used as a positive control. These results indicate that NO-induced GAPDH aggregation directly participates in mitochondrial dysfunction.

One of the most convincing proposed mechanisms underlying mitochondrial swelling and depolarization is the PTP-induced mitochondrial swelling model (39). Based on this model, using cyclosporin A (CsA), which binds to cyclophilin D and inhibits the opening of PTP (39), we examined whether aggregates of GAPDH induce mitochondrial dysfunction via PTP opening. The treatment of isolated mitochondria with aggregates of WT-GAPDH for 30 min elicited mitochondrial swelling and depolarization, whereas these alterations were largely prevented by the addition of CsA (Fig. 3, *F* and *G*). Together, these results suggest that aggregates of GAPDH directly evoke mitochondrial dysfunction, resulting in the swelling and depolarization of mitochondria via the opening of PTP.

##### Aggregates of GAPDH Mediate Mitochondrial Dysfunction in SH-SY5Y Cells

**FIGURE 4.**
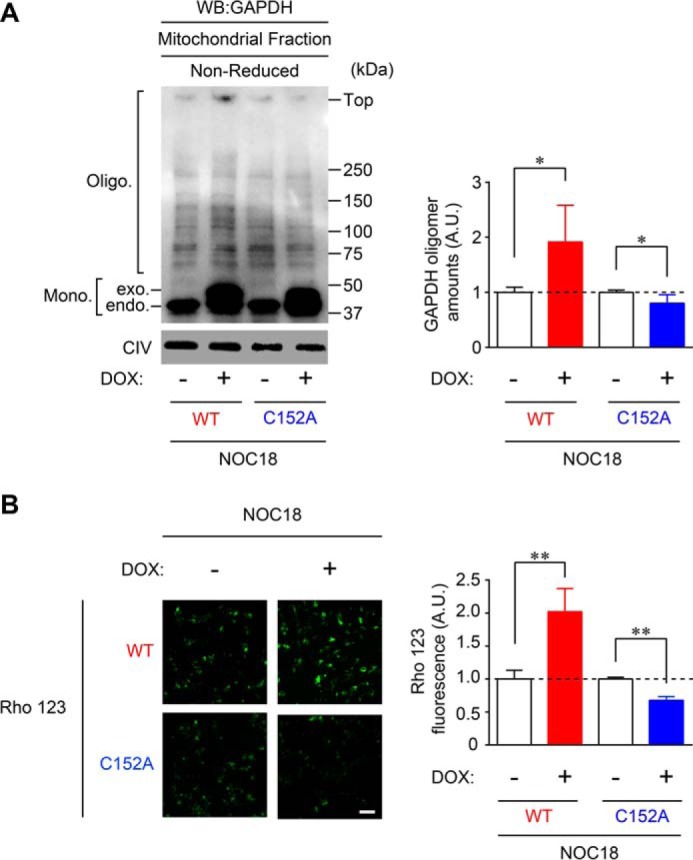
**GAPDH aggregation mediates mitochondrial dysfunction in SH-SY5Y cells.** *A*, effects of overexpression of WT- and C152A-GAPDH on mitochondrial GAPDH aggregation in SH-SY5Y cells. DOX-inducible WT- or C152A-GAPDH-overexpressing cells were treated with NOC18 and then subjected to mitochondrial fractionation followed by Western blotting (*WB*). The amount of GAPDH oligomers was quantified and presented in the *graph* on the *right*. Data are mean ± S.D. (*n* = 4). *, *p* < 0.05, relative to DOX(−), Student's *t* test. *B*, mitochondrial depolarization measured by Rho 123 fluorescence in NOC18-treated WT- or C152A-GAPDH-overexpressing cells. Data are mean ± S.D. (*n* = 4). **, *p* < 0.01, relative to DOX (−), Student's *t* test. *Scale bar*, 100 μm.

To investigate the involvement of aggregates of GAPDH in mitochondrial dysfunction at the cellular level, we used doxycycline (DOX)-inducible WT- or C152A-GAPDH-overexpressing SH-SY5Y cells. Western blotting performed with an anti-GAPDH antibody showed that NOC18-induced GAPDH aggregation in mitochondrial fractions was augmented by overexpression of WT-GAPDH (by 2-fold) but was significantly attenuated by C152A-GAPDH overexpression (by 0.8-fold) (Fig. 4*A*). In addition, NOC18-induced mitochondrial membrane depolarization was measured in these cells by Rho 123 fluorescence (Fig. 4*B*). Similar to the results shown in Fig. 3*D*, NOC18-induced mitochondrial membrane depolarization was significantly increased (by 2-fold) in cells overexpressing WT-GAPDH but inhibited by C152A-GAPDH overexpression (by 0.6-fold) (Fig. 4*B*). These results indicate that NO-induced aggregation of overexpressed GAPDH induces mitochondrial dysfunction in cells.

##### GAPDH Aggregate-mediated Mitochondrial Dysfunction Participates in cyt c Release and Nuclear Translocation of AIF

**FIGURE 5.**
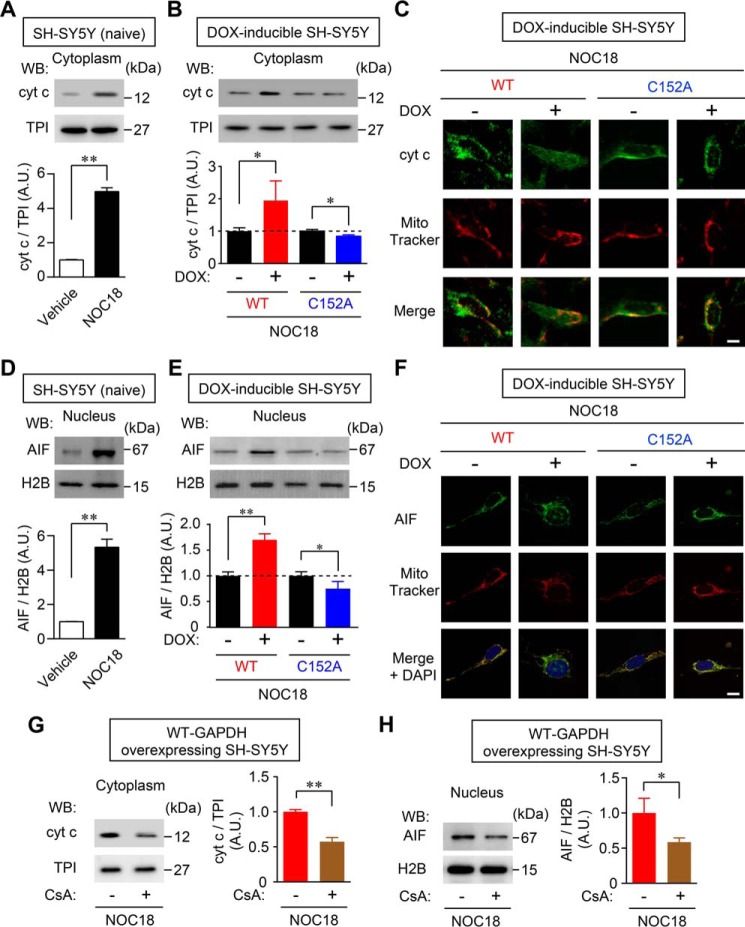
**GAPDH aggregate-mediated mitochondrial dysfunction participates in release of cyt *c* and nuclear translocation of AIF.** *A*, release of cyt *c* into the cytosol of SH-SY5Y cells was induced by treatment with NOC18 for 48 h, as evaluated by cytosolic fractionation and Western blotting (*WB*, *left panel*). The *graph* presents values calculated as the ratio of cyt *c* band intensity relative to TPI band intensity. Data are mean ± S.D. (*n* = 3). **, *p* < 0.01, relative to control, Student's *t* test. *B*, effects of the DOX-induced overexpression of WT- and C152A-GAPDH on cyt *c* release after NOC18 treatment. Data are mean ± S.D. (*n* = 3). *, *p* < 0.05, relative to DOX(−), Student's *t* test. *C*, immunofluorescence of cyt *c* (*green*) and MitoTracker Red (*red*) in DOX-inducible WT- or C152A-GAPDH-overexpressing SH-SY5Y cells treated with NOC18 for 48 h. *Scale bar*, 10 μm. *D*, nuclear translocation of AIF was assessed by Western blotting of nuclear fractions. Values were calculated as the ratio of AIF band intensity to H2B band intensity. Data are mean ± S.D. (*n* = 3). **, *p* < 0.01, relative to vehicle treatment, Student's *t* test. *E*, effects of overexpression of WT- and C152A-GAPDH on nuclear translocation of AIF induced by NOC18 treatment. Data are mean ± S.D. (*n* = 3). *, *p* < 0.05, **, *p* < 0.01, relative to DOX(−), Student's *t* test. *F*, immunofluorescence for AIF (*green*) and MitoTracker Red (*red*) in DOX-inducible WT- or C152A-GAPDH-overexpressing SH-SY5Y cells. Nuclei were stained with DAPI (*blue*). *Scale bar*, 10 μm. *G* and *H*, effect of CsA on NOC18-induced release of cyt *c* and nuclear translocation of AIF, respectively. Data are mean ± S.D. (*n* = 3). *, *p* < 0.05, **, *p* < 0.01, relative to CsA(−), Student's *t* test.

Initiation of cell death induced by oxidative stress from NO involves mitochondrial membrane depolarization and causes the subsequent release of two cell death mediators from the mitochondria: cyt *c*, which goes into the cytosol, and AIF, which translocates into the nucleus (39). Therefore, we investigated whether cyt *c* release into the cytosol and/or nuclear translocation of AIF were caused by GAPDH aggregation. Western blotting showed that when SH-SY5Y cells were treated with NOC18, the levels of cytosolic cyt *c* were significantly increased (Fig. 5*A*). Further, NOC18-induced release of cyt *c* was significantly augmented by the overexpression of WT-GAPDH, whereas the release was decreased by overexpression of C152A-GAPDH (Fig. 5*B*). This result was also observed by immunofluorescence. In the absence of DOX, cyt *c* partially merged with MitoTracker Red. This mitochondrial cyt *c* decreased, likely by diffusion into the cytosol, by overexpression of WT-GAPDH in DOX-treated cells. However, the overlap of MitoTracker Red and cyt *c* was retained by overexpression of C152A-GAPDH (Fig. 5*C*). Similarly, we confirmed that NOC18 induced nuclear translocation of AIF in SH-SY5Y cells (Fig. 5*D*). The levels of AIF in the nuclear fraction from cells treated with NOC18 were significantly increased by overexpression of WT-GAPDH and decreased by overexpression of C152A-GAPDH (Fig. 5*E*). Immunofluorescence showed that overexpression of WT-GAPDH reduced the merging of AIF signals with those of MitoTracker Red and increased nuclear localization of AIF following NOC18 treatment (Fig. 5*F*). Moreover, the merging of AIF and MitoTracker Red signals was retained by overexpression of C152A-GAPDH, which did not induce nuclear translocation of AIF. Additionally, CsA treatment significantly inhibited NOC18-induced cytosolic cyt *c* release (Fig. 5*G*) and AIF nuclear translocation (Fig. 5*H*). These results imply that GAPDH aggregation participates in the cytosolic release of cyt *c* and the nuclear translocation of AIF through PTP-dependent mechanisms under oxidative stress.

##### GAPDH Aggregates Induce Necrotic Cell Death via PTP Opening in SH-SY5Y Cells

**FIGURE 6.**
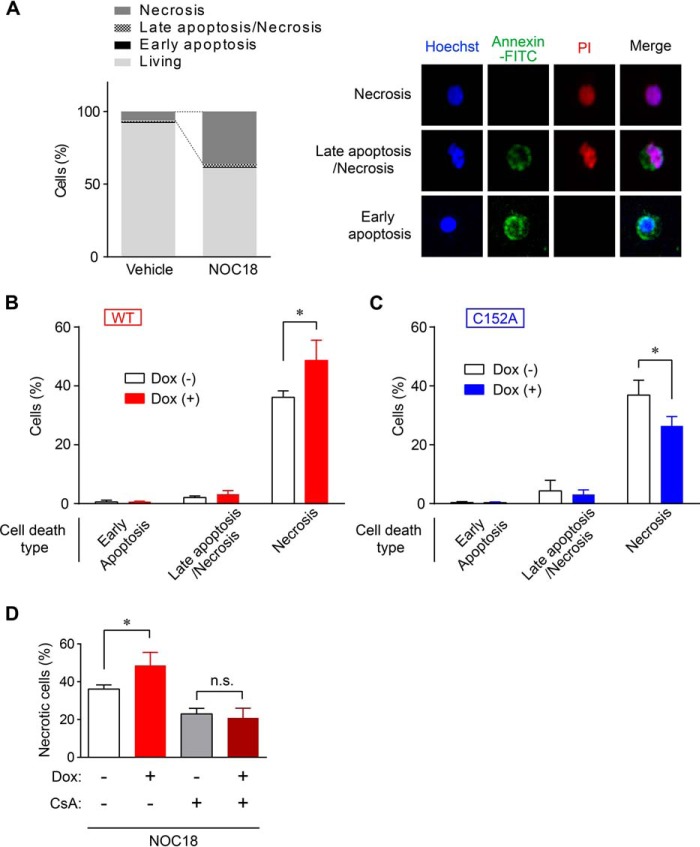
**GAPDH aggregates induce necrosis via the opening of PTP.** *A*, NOC18-induced ell death type was examined by PI/annexin V-FITC staining (*left graph*). Representative images of necrosis, late apoptosis/necrosis, and early apoptosis are shown in the *right panels. B* and *C*, effect of overexpression of WT- or C152A-GAPDH on type of cell death (early apoptosis, late apoptosis/necrosis, or necrosis). Data are mean ± S.D. (*n* = 3). *, *p* < 0.05, relative to DOX(−), Student's *t* test. *D*, effect of CsA on NOC18-induced necrotic cell death in DOX-inducible WT- GAPDH-overexpressing SH-SY5Y cells. Data are mean ± S.D. (*n* = 3). *, *p* < 0.05, relative to DOX(−); *n.s*., not significant by Student's *t* test.

We next evaluated the type of cell death induced by GAPDH aggregates using propidium iodide (PI)/annexin V staining. A large percentage of cell death induced by NOC18 treatment was necrotic (about 36%, PI+/annexin V− cells), whereas early apoptosis (PI−/annexin V+ cells) and late apoptosis/necrosis (PI+/annexin V+ cells) were hardly seen (Fig. 6*A*). To determine the type of cell death induced by GAPDH aggregates, we employed either WT- or C152A-GAPDH-overexpressing cells. Necrosis was significantly increased in cells overexpressing WT-GAPDH but was decreased by C152A-GAPDH overexpression (Fig. 6, *B* and *C*). Notably, there were no significant differences in other types of cell death (Fig. 6, *B* and *C*). Greater necrosis following WT-GAPDH overexpression was completely abolished by CsA treatment (Fig. 6*D*), indicating that NOC18-induced GAPDH aggregates cause necrotic cell death via PTP opening.

##### GAPDH Aggregation Does Not Affect Oxidative Stress-induced Proteasome Activity, ER Stress-related Protein Expression, or Autophagy

**FIGURE 7.**
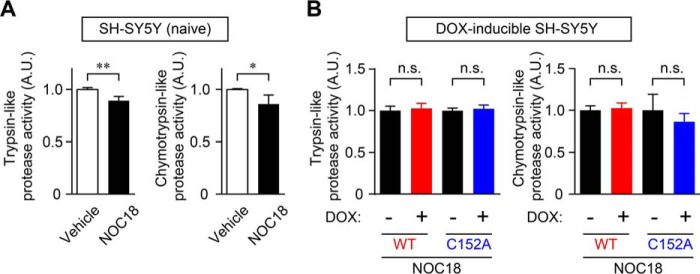
**Proteasome activity is not affected by GAPDH aggregation.** *A*, reduction of trypsin-like and chymotrypsin-like protease activities caused by the treatment of SH-SY5Y cells with NOC18 for 48 h. Data are mean ± S.D. (*n* = 4). *, *p* < 0.05, **, *p* < 0.01, relative to vehicle treatment, Student's *t* test. *B*, effects of the overexpression of WT- and C152A-GAPDH on trypsin-like and chymotrypsin-like protease activities with NOC18 treatment. Data are mean ± S.D. (*n* = 4). *n.s*., not significant by Student's *t* test. *A.U.* indicates arbitrary units.

In addition to mitochondrial dysfunction, several lines of evidence suggest that the proteasome is also involved in the toxicity of amyloidogenically aggregated proteins, such as amyloid-β and α-synuclein, and these aggregated proteins attenuate the activity of proteasomes in neurons and contribute to cell death (38, 40). Therefore, we studied the effects of GAPDH aggregation on trypsin- and chymotrypsin-like proteasome activities (Fig. 7). We first confirmed the decrease of proteasome activities in NOC18-treated SH-SY5Y cells (Fig. 7*A*). However, the decline of these proteasome activities induced by NOC18 treatment was not altered by overexpression of either WT- or C152A-GAPDH (Fig. 7*B*).

**FIGURE 8.**
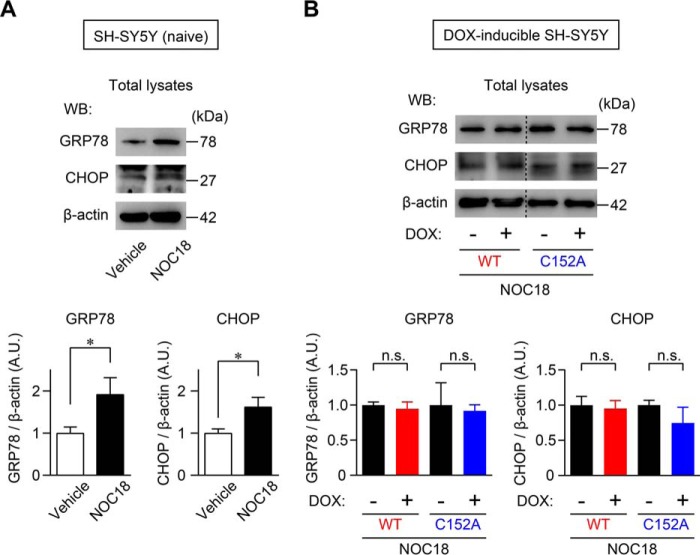
**Endoplasmic reticulum stress-related protein expression is not affected by GAPDH aggregation.** *A*, up-regulation of GRP78 and CHOP in SH-SY5Y cells treated with NOC18 for 48 h, as evaluated by Western blotting. The *graphs* present the ratios of GRP78 or CHOP band intensity relative to β-actin band intensity. Data are mean ± S.D. (*n* = 3). *, *p* < 0.05, relative to vehicle treatment, Student's *t* test. *B*, effects of overexpression of WT- and C152A-GAPDH on the expression of GRP78 and CHOP with NOC18 treatment. Data are mean ± S.D. (*n* = 3); *n.s*., not significant by Student's *t* test. *A.U.* indicates arbitrary units.

We also evaluated the influence of GAPDH aggregation on ER stress, which is considered critical for aggregated protein-induced cell death, by assessing the expressions of the ER chaperone glucose-regulated protein 78 (GRP78; also called KDEL) and the ER-related proapoptotic protein C/EBP homologous protein (CHOP) (41, 42). An up-regulation of the GRP78 and CHOP protein levels was observed in NOC18-treated SH-SY5Y cells (Fig. 8*A*), which was not altered in cells overexpressing WT- or C152A-GAPDH (Fig. 8*B*).

**FIGURE 9.**
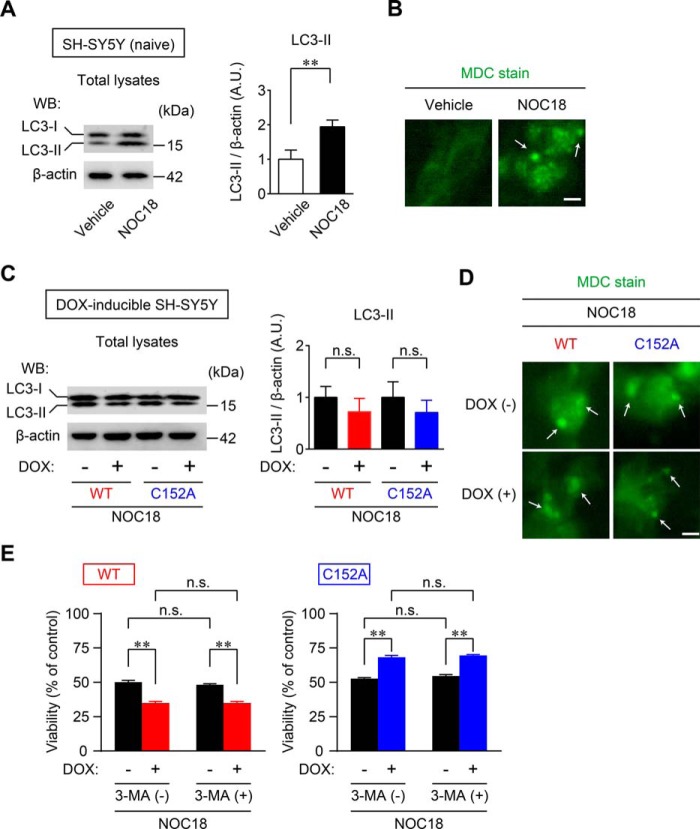
**Induction of autophagy is not affected by GAPDH aggregation.** *A*, NOC18-induced autophagy was measured by the conversion of LC3-I (18 kDa) to LC3-II (16 kDa) as assessed by Western blotting (*WB*). The *graph* presents values calculated as the ratio of LC3-II band intensity to β-actin band intensity. Data are mean ± S.D. (*n* = 3). **, *p* < 0.01, relative to vehicle treatment, Student's *t* test. *B*, fluorescence of MDC staining of NOC18-treated SH-SY5Y cells. *Arrows* show punctate signals, indicating the induction of autophagy. *Scale bar*, 10 μm. *C*, effects of overexpression of WT- and C152A-GAPDH on the induction of autophagy with NOC18 treatment as evaluated by Western blotting. Data are mean ± S.D. (*n* = 3). *D*, fluorescence of MDC staining in DOX-inducible WT- and C152A-GAPDH-overexpressing SH-SY5Y cells with NOC18 treatment. *Arrows* show MDC-positive vesicles, indicating the induction of autophagy. *Scale bar*, 10 μm. *E*, effects of autophagy inhibitor 3-MA (1 mm) on the cell viability in WT (*left graph*)- or C152A (right graph)-GAPDH-overexpressing SH-SY5Y cells treated with NOC18 for 48 h. Data are mean ± S.D. (*n* = 4). **, *p* < 0.01, relative to DOX(−), Student's *t* test. *A.U.* indicates arbitrary units.

Lastly, we examined the effects of GAPDH aggregation on autophagy. In SH-SY5Y cells, the induction of autophagy by treatment with NOC18 was confirmed by modification of microtubule-associated protein 1 light chain 3B (LC3), observed as an increase in the amount of the faster migrating form, LC3-II (Fig. 9*A*). The conversion from LCS-I to LCS-II is considered a marker of autophagy (43). Concomitantly, the induction of autophagy was morphologically ascertained by the presence of punctate structures stained with monodansylcadaverine (MDC) (Fig. 9*B*), which accumulates in autophagic vacuoles (43). By contrast, neither the increase in LC3-II nor the presence of MDC-positive vacuoles was altered by overexpression of WT- and C152A-GAPDH (Fig. 9, *C* and *D*). Additionally, the viability of WT- and C152A-GAPDH-overexpressing cells was tested in the presence or absence of 3-methyladfenine (3-MA), an autophagy inhibitor. Similar to results from our previous report (24), we observed exacerbation and amelioration of cell viability under NOC18 treatment following overexpression of WT- and C152A-GAPDH, respectively; these effects were not observed upon treatment with 3-MA (Fig. 9*E*). Together, these observations indicate that proteasome activity, ER stress-related protein expression, and autophagy do not contribute to mechanisms that mediate GAPDH aggregate-induced cell death with NOC18 treatment.

## Discussion

In this study, we have demonstrated that NO induced the formation of GAPDH aggregates at mitochondria, leading to mitochondrial dysfunction (Figs. 1 and 2). Moreover, experiments using GAPDH-free isolated mitochondria showed that GAPDH aggregates directly induced mitochondrial swelling and depolarization via PTP opening (Fig. 3). Additionally, reducing GAPDH aggregation by expressing a dominant-negative mutant, C152A-GAPDH, ameliorated NO-induced mitochondrial depolarization (Fig. 4), subsequent cytosolic release of cyt *c* and nuclear translocation of AIF (Fig. 5), and necrotic cell death in a PTP-dependent manner (Fig. 6). By contrast, NO-induced alterations of proteasome activity, ER stress-related protein expression, and autophagy were not affected by GAPDH aggregation (Figs. 7–9). These results suggest a crucial role for GAPDH aggregation in mitochondrial dysfunction in cells under oxidative stress.

NO-induced GAPDH aggregation appears to be composed of mitochondrial GAPDH. Our results show that under treatment with the NO generator NOC18 at a concentration leading to 50% reduction in cell viability, aggregates of GAPDH emerged along with mitochondrial dysfunction (Fig. 1). Furthermore, NO-induced aggregates of GAPDH localized to the mitochondria; these aggregates were derived from mitochondrial GAPDH rather than recruited from other organelles (Fig. 2). In contrast, it was reported that GAPDH translocates to the mitochondria of cerebellar granule cells and several tumor cell lines during apoptotic stimulation, such as with serum deprivation or treatments with staurosporine, etoposide, and lonidamine (35). This difference may be due to the type of stimulus. Indeed, aggregates of GAPDH are generated by various stimuli, such as hydrogen peroxide, dopamine, peroxynitrite, and *S*-nitrosoglutathione (13), indicating that a variety of mechanisms underlie mitochondrial dysfunction.

Aggregates of GAPDH directly induce mitochondrial dysfunction. In our experiments using isolated mitochondria free from endogenous GAPDH, treatment with aggregates of GAPDH led to mitochondrial swelling and depolarization. These effects were attenuated by the addition of C152A-GAPDH, which reduces the amount of aggregation (Fig. 3). Alternatively, treatment with soluble GAPDH did not evoke mitochondrial dysfunction (data not shown), which contradicts the findings of Tarze *et al.* (35). However, their findings (35) were obtained from commercially available GAPDH. It is known that GAPDH is prone to aggregate unless prepared with a reducing reagent such as DTT (14, 15). It is possible that the GAPDH (25 μg/ml) used in their study was already aggregated. Indeed, we found that with a high concentration (100 μg/ml) of soluble GAPDH prepared using our established procedure for purification of fully reduced GAPDH, which can trigger GAPDH aggregation even in the absence of NOC18, induced mitochondrial dysfunction (data not shown). These observations suggest that aggregated GAPDH has more deleterious effects on mitochondria than does soluble GAPDH.

How do aggregates of GAPDH cause mitochondrial dysfunction? Our data from isolated CsA-treated mitochondria indicate that GAPDH aggregate-induced mitochondrial swelling and depolarization are due to PTP opening (Fig. 3, *F* and *G*). Several studies have reported on the mechanisms of mitochondrial dysfunction induced by other aggregated proteins such as amyloid-β, α-synuclein, and mutant huntingtin via PTP (44–46). However, exactly how aggregated protein could open the PTP is not clear. *S*-Nitrosylated GAPDH reportedly acts as a mitochondrial trans-*S*-nitrosylase in the heart (47). Hence, to explore the influence of the trans-*S*-nitrosylase activity of GAPDH aggregates, we first determined the *S*-nitrosothiols (SNO) of GAPDH aggregates using Saville's method described in Ref. 48. The result indicated that GAPDH aggregates have almost no *S*-nitrosothiols (0.10 ± 0.02 SNOs/tetramer), similar to soluble GAPDH (0.10 ± 0.01 SNOs/tetramer (supplemental Fig. S1*A*)). To further investigate the ability of GAPDH aggregate to act as a mitochondrial trans-*S*-nitrosylase, SNO levels of mitochondrial proteins were examined using isolated mitochondria and the biotin switch method. Compared with mitochondria incubated with vehicle, those treated with GAPDH aggregates did not show different SNO-protein band patterns (supplemental Fig. S1*B*). Although it is possible that the trans-*S*-nitrosylase activity of GAPDH may influence intracellular mitochondrial function, taking into consideration that GAPDH aggregates directly cause dysfunction in isolated mitochondria (Fig. 3), the association of a mechanism other than trans-*S*-nitrosylation could be considered.

Mitochondrial GAPDH aggregation may be a key regulator of NO-induced necrotic cell death concomitant with the release of cyt *c* and AIF from the mitochondria. Our data from DOX-inducible WT- or C152A-GAPDH-overexpressing SH-SY5Y cells demonstrate that the amount of mitochondrial GAPDH aggregation correlates with mitochondrial dysfunction resulting in cyt *c* release into the cytosol and nuclear translocation of AIF (Figs. 4 and 5). Cyt *c* release is thought to occur via permeability transition-associated mitochondrial swelling and the subsequent rupture of the outer mitochondrial membrane (49). Similarly, nuclear translocation of AIF from the mitochondrial intermembrane occurs concurrently with the disruption of mitochondrial membrane potential (50). These findings support our results showing that PTP inhibition by CsA treatment suppressed cytosolic cyt *c* release and nuclear translocation of AIF (Fig. 5, *G* and *H*). Interestingly, a large population of cell death induced by GAPDH aggregates was necrotic (Fig. 6), even though both cyt *c* and AIF are generally regarded as apoptotic molecules. Severe ATP depletion and plasma membrane integrity loss are primary drivers of necrosis, but activation of downstream apoptotic signaling is also thought to be a contributor (51). There is growing evidence that AIF is the key molecule in programmed necrosis (termed “necroptosis”) (52). Hence, there is a need for further investigation into the contributions of cyt *c* and/or AIF to GAPDH aggregate-induced necrosis.

It is well known that aggregates of amyloidogenic protein are involved in proteasome activity (38) and ER stress (41, 42). Aggregates of GAPDH may also contribute to the proteasome inhibition and ER stress responsible for NO-induced cell death (53, 54), as the aggregates in our study were not strictly localized to the mitochondria (Fig. 2*B*). However, our results indicate that neither NO-induced proteasome inhibition nor ER stress is affected by altering the amount of GAPDH aggregation (via overexpression of WT- and C152A-GAPDH (Figs. 7 and 8)). In addition, autophagy has an important role in NO-induced cell responses (55) and is regulated by GAPDH expression via an increase of Atg12 expression (56). Nonetheless, although NO stress induced autophagy, it was not mediated by the amount of GAPDH aggregation (Fig. 9).

Nuclear translocation of GAPDH is not likely to participate in the death of SH-SY5Y cells treated with NOC18. We assessed whether WT- or C152A-GAPDH overexpression influences the nuclear translocation of GAPDH induced by NOC18-treatment (supplemental Fig. S2). Treatment of naive SH-SY5Y with NOC18 induced distinct nuclear translocation of GAPDH (supplemental Fig. S2, *A* and *B*). The amount of nuclear GAPDH was increased by overexpressing WT-GAPDH but not by overexpressing C152A-GAPDH (supplemental Fig. S2, *C* and *D*). Further, we assessed the viability of WT- and C152A-GAPDH-overexpressing cells in the presence or absence of deprenyl, an inhibitor of GAPDH nuclear translocation, and showed that nuclear translocation of GAPDH does not regulate cell viability (supplemental Fig. S2*E*). Consequently, these results support those from our previous study showing that high levels of oxidative stress are unrelated to the nuclear translocation of GAPDH (14).

**FIGURE 10.**
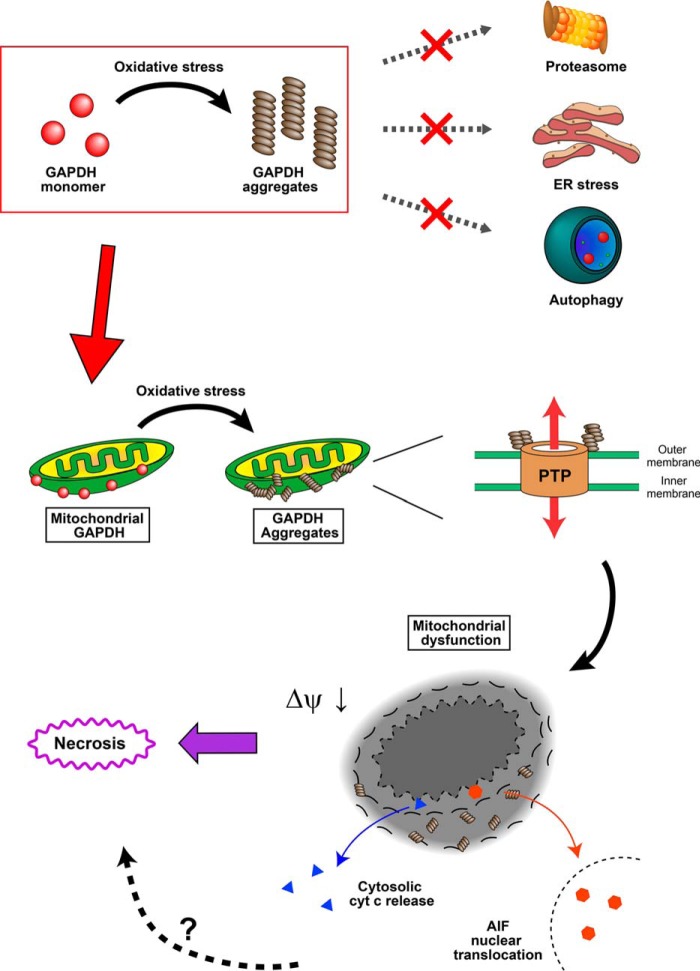
**Schema of GAPDH aggregate-induced mitochondrial dysfunction.** Aggregates of GAPDH are formed at mitochondria, which directly induce a decrease in mitochondrial membrane potential (Δψ) and mitochondrial swelling via the opening of a PTP. Mitochondrial dysfunction results in both cytosolic release of cyt *c* and nuclear translocation of AIF, leading to necrosis. Meanwhile, aggregates of GAPDH do not influence proteasome activity, the ER stress cascade, or the induction of autophagy.

In summary, the present study demonstrates that NO-induced aggregates of GAPDH mediate mitochondrial dysfunction and subsequent necrotic cell death concomitant with cyt *c* release and AIF nuclear translocation (Fig. 10). It is well recognized that NO has critical roles in the pathogeneses of several diseases, such as Alzheimer's disease, Parkinson's disease, and stroke (57). Interestingly, there is some evidence suggesting the involvement of GAPDH aggregation in Alzheimer's and Parkinson's diseases (18, 21, 23). We have also found that aggregates of GAPDH form abundantly after middle cerebral artery occlusion in a mouse stroke model in which oxidative stress is responsible for neuronal cell death (in preparation). Thus, the findings in the present study could help in understanding the molecular mechanism underlying oxidative stress-related diseases and provide new therapeutic approaches for brain damage.

## Experimental Procedures

### 

#### 

##### Chemicals, Antibodies, and Plasmids

Unless otherwise noted, the chemicals used were of analytical grade. Antibodies were as follows: mouse monoclonal anti-GAPDH (MAB374) and rabbit polyclonal anti-histone H2B (catalog No. 07-371) from Millipore; rabbit polyclonal anti-AIF (AF1475) from R&D Systems; mouse monoclonal anti-cyt *c* (catalog No. 556433) from BD Biosciences; rabbit polyclonal anti-Myc (A-14, sc-789), rabbit polyclonal anti-CHOP (F-168, sc-575), and rabbit polyclonal anti-His (Omni-probe M-21, sc-499) from Santa Cruz Biotechnology; mouse monoclonal anti-CIV (1D6E1A8) from Invitrogen; monoclonal anti-KDEL (GRP78) (10C3) from Stressgen; monoclonal anti-β-actin (AC15) from Sigma; mouse monoclonal anti-LC3 (2G6) from NanoTools; and polyclonal anti-triosephosphate isomerase (TPI) prepared in house (58).

The cloning of human WT GAPDH cDNA was performed as reported previously (4). For bacterial expression, cDNA was cloned into pBAD-HisA (Invitrogen) using the SacI and KpnI sites. For mammalian cell line expression, the cDNA was cloned into pcDNA4-TO-Myc/HisA (Invitrogen) using the EcoRI and EcoRV sites. Using WT-GAPDH as a template, the alanine-substituted mutant C152A-GAPDH was generated with the QuikChange site-directed mutagenesis kit (Stratagene) according to the manufacturer's protocol, as reported previously (15). 3-MA was purchased from Sigma. Deprenyl was kindly provided by Fujimoto Pharmaceutical (Osaka, Japan).

##### Cell Culture and Cell Viability

Human neuroblastoma SH-SY5Y cells (ATCC) were grown in DMEM/F12 supplemented with 10% FBS, 2 mm glutamine, and antibiotics-antimycotics (Invitrogen) at 37 °C in a 5% CO_2_ humidified incubator. Generation of stable cell lines for inducible expression of GAPDH was established as reported previously (15). SH-SY5Y cells were cotransfected with pcDNA6/TR (Invitrogen) and pcDNA4-TO/Myc-HisA vectors carrying WT- or C152A-GAPDH using Lipofectamine 2000 (Invitrogen) or HilyMax (Dojindo). Stable cells resistant to both blasticidin (20 μg/ml) and zeocin (100 μg/ml) were cultivated. Inducible expression of Myc-tagged GAPDH was performed by treatment with DOX (1 μg/ml) for 3–6 days. Cell viability was measured using the CellTiter-Glo luminescent cell viability assay kit (Promega) according to the manufacturer's protocol (15).

##### Subcellular Fractionations

Subcellular fractionations were carried out according to the following procedures. After a 48-h treatment with either control vehicle (0.1 n NaOH) or the NO generator NOC18 (Dojindo), cells were washed twice with PBS and then incubated for 5 min in ice-cold PBS containing 40 mm iodoacetamide to protect unmodified thiols from oxidation during fractionation. All subsequent steps were performed at 4 °C. For isolation of the insoluble fraction, cells were scraped in 500 μl of buffer A (10 mm Tris-HCl, pH 7.5, 10 mm NaCl, 3 mm MgCl_2_, 0.05% Nonidet P-40, 0.5% Triton X-100, 40 mm iodoacetamide, 1 mm PMSF, and protease inhibitor mixture (Roche Diagnostics)). After 30 min, the suspensions of cells were homogenized vigorously for 15 s with rocking. The total lysates were centrifuged at 20,400 × *g* for 10 min, and both the total cell lysates and pellets were collected to prepare insoluble fractions. To detect insoluble GAPDH oligomers in cells, the pellets obtained from total cell lysates were resuspended in 200 μl of buffer B (10 mm HEPES-KOH, pH 7.4, 25 mm NaCl, 3 mm MgCl_2_, 300 mm sucrose, 40 mm iodoacetamide, 1 mm PMSF, and protease inhibitor mixture (Roche Diagnostics)), washed three times by centrifugation (3000 × *g* for 10 min), and again resuspended in the buffer. After the addition of 100 μl of buffer B, the pellets were sonicated for 30 s and finally obtained as insoluble fractions. These fractions were stored at −80 °C for further use. Protein concentrations of the samples were determined using the Bradford assay (Bio-Rad).

Mitochondria were isolated from cells as reported previously (37) with some modifications. Briefly, after treatments with or without 200 μm NOC18 for 48 h, cells were washed twice with PBS and then homogenized in isolation buffer (5 mm HEPES-KOH, pH 7.5, 210 mm mannitol, 70 mm sucrose, 1 mm EDTA, and 110 μg/ml digitonin). Homogenates were centrifuged at 5000 × *g* for 20 min at 4 °C, and then the cell pellet was resuspended in isolation buffer. The suspensions were homogenized and centrifuged (2000 × *g* for 5 min at 4 °C). Supernatants were further centrifuged at 11,000 × *g* for 10 min at 4 °C, and the resultant pellets were used as mitochondrial fractions.

##### Semiquantification of Mitochondrial Depolarization

This procedure was performed as described previously (59) with minor modifications. Cells were treated with 5 μm Rho 123 for 30 min at 37 °C with 5% CO_2_. Cells were washed with PBS and stained with Hoechst 33342. The fluorescence of Rho 123 was detected by a confocal scanning microscope (C1si-TE2000-E, Nikon, Japan). For semiquantification of fluorescence of Rho 123, four to seven microscopic fields were selected randomly, and the fluorescence values (excitation wavelength, 485 nm; emission wavelength, 535 nm) were measured using an Ez-C1 free viewer (Nikon). Concomitantly, the total number of cells stained with Hoechst 33342 (2 μg/ml) in the same field was counted, and substantial values of fluorescence of Rho 123 were normalized.

##### Assessment of GAPDH Aggregation

To detect aggregates of GAPDH in insoluble and mitochondrial fractions, each fraction was mixed with low SDS sample buffer (final concentration: 62.5 mm Tris-HCl, pH 6.8, 0.5% SDS, 10% glycerol, and 0.002% bromphenol blue) and then heated at 100 °C for 5 min. These samples were separated by 5–20% non-reducing SDS-PAGE and transferred to a nitrocellulose membrane (Bio-Rad). The membranes were incubated for 1 h with Blocking One (Nacalai Tesque) to block nonspecific binding. The membrane was then incubated for 2 h at room temperature with an anti-GAPDH monoclonal antibody (1:300) in 10% Blocking One-PBST (0.05% Tween 20 and 0.02% NaN_3_ in PBS) followed by incubation for 1 h at room temperature with a peroxidase-conjugated affinity-purified secondary antibody (Invitrogen). The signals were detected using both SuperSignal West Pico chemiluminescent substrate (GE Healthcare) and LAS3000 (Fujifilm, Tokyo). To assess the purity of mitochondria isolated from cells, Western blotting was performed using an anti-CIV monoclonal antibody (1:1000, a mitochondrial marker), an anti-histone H2B polyclonal antibody (1:5000, a nuclear marker), or an anti-TPI polyclonal antibody (1:1000, a cytosolic marker); membranes were also blotted with an anti-GAPDH antibody.

##### Cell Immunofluorescence

Immunofluorescence we performed as described previously (4) with minor modifications. For mitochondria staining, cells were treated with MitoTracker Red CMXRos at 250 nm (Life Technologies) for 30 min at 37 °C with 5% CO_2_. Cells were then washed with PBS and fixed with 4% paraformaldehyde in PBS, pH 7.4, for 10 min at room temperature. The cells were permeabilized for 5 min with PBS containing 0.1% Triton X-100 and incubated with 10% BSA in PBS for 1 h at room temperature. The cells were incubated overnight at 4 °C with an anti-GAPDH (1:1000), anti-AIF (1:10,000), or anti-cyt *c* (1:200) antibodies in 10% Blocking One-PBST. After three washes with PBST, the specific signal was visualized by staining the cells with an Alexa Fluor 568-conjugated secondary antibody (1:1000; Invitrogen) using a confocal scanning microscope (C1si-TE2000-E, Nikon).

##### Expression and Purification of Recombinant GAPDH

The pBAD-HisA vector carrying WT- or C152A-GAPDH cDNA was transformed into GAP(−) *Escherichia coli* strain W3CG (60). Expression and purification of these recombinant GAPDH proteins were carried out as described previously (14, 15). Protein concentrations were determined spectrophotometrically assuming ϵ0.1% at 280 nm = 1.0.

##### Preparation of Aggregates of GAPDH

The aggregates of GAPDH were prepared as described in the published protocols (24). Briefly, purified recombinant WT-GAPDH (0.3 mg/ml) with or without C152A-GAPDH (0.75 mg/ml) was treated for 24 h with 100 μm NOR3 ((±)-(*E*)-4-ethyl-2-[(*E*)-hydroxyimino]-5-nitro-3-hexenamide) in G2′ buffer (50 mm Tris-HCl, pH 8.0, 150 mm NaCl, 1 mm EDTA, and 5% glycerol). The prepared aggregates were applied immediately to various experiments in the present study.

##### Preparation of Isolated Mitochondria from Mice

Liver mitochondria from mice (C57BL/6J males, 20–30 g, SLC Japan) were isolated as reported previously (40) with some modifications. Briefly, the liver was diced and then homogenized in solution A, pH 7.4 (10 mm Tris-HCl, 0.25 m sucrose, and 0.1 mm EDTA). After centrifugation at 80 × *g* for 7 min, the supernatant was layered on the same amount of solution B, pH 7.4, containing 10 mm Tris-HCl, 0.35 m sucrose, and 0.1 mm EDTA and centrifuged at 700 × *g* for 10 min. The supernatant was centrifuged at 7000 × *g* for 10 min. The pellet was resuspended in solution C, pH 7.4 (10 mm Tris-HCl and 0.25 m sucrose), and centrifuged again at 7000 × *g* for 10 min. The final mitochondrial pellet was resuspended in solution C. The solution containing the purified mitochondria was used immediately.

##### Transmission Electron Microscopy

Isolated mitochondria were treated with aggregates of GAPDH for 30 min at room temperature and then centrifuged at 7000 × *g* for 10 min. The pellet containing the mitochondria was fixed in 2.5% buffered glutaraldehyde and postfixed in 1% buffered osmium tetroxide. The samples were gradually dehydrated and then embedded in epoxy resin for 48 h at 60 °C. Thin sections (80 nm) stained with uranyl acetate and lead citrate were examined with an electron microscope (Hitachi H-7500) at 75 kV. A mitochondrial cross-sectional area was quantified by using ImageJ software (National Institutes of Health, Bethesda, MD).

##### Measurement of GAPDH Enzyme Activity

An assay solution (50 mm triethanolamine, pH 8.9, 0.2 mm EDTA, and 50 mm K_2_PO_4_) was mixed with 1 mm NAD^+^ and 10 μg of sample. The enzyme activity was initiated by the addition of 2 mm glyceraldehyde-3-phosphate. GAPDH activity was measured for 1 min at 25 °C via the change in absorbance at 340 nm using a VERSAmax microplate reader (Molecular Devices, Tokyo).

##### In Vitro Mitochondrial Swelling Assay and Mitochondrial Depolarization Assay

The mitochondrial swelling assay was carried out as described previously (61). The isolated mitochondria were diluted at 0.2 mg/ml in buffer M, pH 7.4 (10 mm Tris-HCl, 10 mm Mops, 5 mm succinate, 200 mm sucrose, 1 mm P_i_, 10 μm EGTA, and 2 μm rotenone). After adding aggregates of GAPDH, mitochondrial swelling was measured by the decrease in absorbance at 540 nm for 150 min. The mitochondrial depolarization assay was performed as described previously (62). The isolated mitochondria were diluted at 0.2 mg/ml in buffer M and mixed with Rho 123 (5 μm). After a 5-min incubation, aggregates of GAPDH were added, and the fluorescence (excitation wavelength, 485 nm; emission wavelength, 535 nm) was measured using an ARBO multilabel plate reader (PerkinElmer Life Sciences). CsA (1 μm) was added 2 min before treatment with GAPDH aggregates for PTP inhibition.

##### Examination of Cytosolic Cyt c and Nuclear AIF

Cells were treated with or without 200 μm NOC18 for 48 h, washed twice with PBS, and then incubated for 10 min in ice-cold hypotonic buffer (10 mm Tris-HCl, pH 7.4, 10 mm NaCl, 0.05% Nonidet P-40, 1 mm PMSF, and protease inhibitor). CsA (1 μm) was added 1 h before treatment with NOC18 for PTP inhibition. The lysates were centrifuged at 3200 × *g* for 5 min, and the supernatants and pellets were collected. The supernatants were further centrifuged at 100,000 × *g* for 60 min, and these supernatants were obtained as the cytosolic fraction. The pellets were suspended in isotonic buffer (10 mm HEPES-KOH, pH 7.5, 25 mm NaCl, 300 mm sucrose, 1 mm PMSF, and protease inhibitor), washed twice followed by centrifugation at 3200 × *g* for 1 min, and resuspended in the buffer. After the addition of hypertonic buffer (50 mm HEPES-KOH, pH 7.5, 400 mm KCl, 3 mm MgCl_2_, 1 mm EDTA, 1 mm DTT, 0.5% Triton X-100, 0.05% Nonidet P-40, 0.2 mm PMSF, and protease inhibitor), the pellets were sonicated for 30 s and obtained as the nuclear fraction. Western blotting was performed as described above using polyclonal anti-AIF (1:10,000) and monoclonal anti-cyt *c* (1:500) antibodies. The membranes were also reprobed with an anti-TPI antibody (1:4000) as a cytosolic marker or an anti-histone H2B polyclonal antibody (1:5000) as a nuclear marker.

##### Apoptosis/Necrosis Assay

The apoptosis assay was conducted using the MEBCYTO apoptosis kit (MBL, Nagoya, Japan) according to the manufacturer's instructions with minor modifications. In brief, cells were washed and resuspended in binding buffer. Hoechst 33342 (2 μg/ml), annexin V-FITC, and PI were added to the cell suspension, and then the mixture was incubated for 15 min in the dark at room temperature. Thereafter, the suspension was analyzed using a confocal scanning microscope (C1si-TE2000-E, Nikon). Quantitative analysis was performed by counting more than 700 cells in each examination.

##### Proteasome Activity

This assay was performed as described previously (63) with minor modifications. Proteasome activity was detected by hydrolysis of the fluorogenic peptide Boc-LRR-MCA (for trypsin-like protease activity; Peptide Institute, Inc.) or Suc-LLVY-MCA (for chymotrypsin-like protease activity; Peptide Institute, Inc.). Briefly, cells were washed twice with PBS and lysed by freezing for 15 min at −80 °C and thawing for 15 min at 37 °C in lysis buffer (20 mm Tris-HCl, pH 7.4, 0.1 mm EDTA, 1 mm 2-mercaptoethanol, 5 mm ATP, 20% glycerol, and 0.04% Nonidet P-40). Freeze-thaw cycles were repeated four times, and lysates were centrifuged at 13,000 × *g* for 15 min at 4 °C. Protein concentrations of the samples were determined by the Bradford assay. The reaction mixture contained 50 mm HEPES-KOH, pH 8.0, 5 mm EGTA, and 100 μm Boc-LRR-MCA or Suc-LLVY-MCA. The reaction was performed at 37 °C for 60 min and stopped by the addition of 1% SDS. The fluorescence was detected by an ARBO multilabel plate reader (PerkinElmer Life Sciences) at an excitation wavelength of 380 nm and emission wavelength of 440 nm.

##### Detecting Levels of Major ER Stress Proteins

Cells were washed twice with PBS and lysed in buffer (50 mm Tris-HCl, pH 7.5, 0.5% Nonidet P-40, 150 mm NaCl, 1 mm EDTA, 1 mm PMSF, 100 μm sodium vanadate, 1 mm sodium fluoride, and protease inhibitor mixture). After rotating for 30 min at 4 °C, the lysates were centrifuged at 20,400 × *g* for 15 min at 4 °C, and the supernatants were subjected to Western blotting as described above using monoclonal anti-KDEL (GRP78), polyclonal anti-CHOP, and monoclonal anti-β actin (internal control) antibodies.

##### Examination of NO-induced Autophagy

For the detection of LC3, cells were lysed in lysis buffer (50 mm Tris-HCl, pH 7.5, 1% Triton X-100, 150 mm NaCl, and protease inhibitor) and centrifuged at 16,400 × *g* for 20 min. The supernatants were subjected to SDS-PAGE and transferred to PVDF membranes. Western blotting was carried out with anti-LC3 (1:1000) (kindly supplied by Dr. Matsuzawa, Osaka Prefecture University) and anti-GAPDH antibodies. For MDC staining, the cells were treated with either vehicle or NOC18 and then stained with 200 μm MDC for 45 min. The cells were washed with PBS, and fluorescence was detected as described for cell immunofluorescence.

##### Statistical Analysis

All data are presented as the mean ± S.D. of independent experiments as indicated (*n*) in each figure legend (Figs. 1–9). For statistical analysis, two or multiple groups were compared with unpaired Student's *t* tests or Dunnett's multiple tests after one-way analysis of variance, respectively.

## Author Contributions

H. N. designed the study. M. I., T. K., A. K., N. H., R. Y., and H. N. performed the biochemical and cell-based studies. M. I., T. I., and M. K. performed the transmission electron microscopy. M. I., T. K., H. N., A. K., Y. A., and R. Y. analyzed the data. H. N. and M. I. wrote the paper, and H. N. and T. T. supervised the study.

## Supplementary Material

Supplemental Data

## Footnotes

3 The abbreviations used are:

ER: endoplasmic reticulum
3-MA: 3-methyladfenine
AIF: apoptosis-inducing factor
CHOP: C/EBP homologous protein
CIV: cytochrome *c* oxidase complex IV
CsA: cyclosporin A
cyt *c*: cytochrome *c*
DOX: doxycycline
GRP78: glucose-regulated protein 78
LC3: light chain 3B
MDC: monodansylcadaverine
NOC18: 1-hydroxy-2-oxo-3,3-bis(2-aminoethyl)-1-triazene
PI: propidium iodide
PTP: permeability transition pore
Rho 123: rhodamine 123 (6-amino-9-[2-(methoxycarbonyl)phenyl]-3*H*-xanthen-3-iminium chloride)
SNO: *S*-nitrosothiol
TPI: triosephosphate isomerase.
