# Supplementary material for: Glyceraldehyde-3-phosphate Dehydrogenase (GAPDH) Aggregation Causes Mitochondrial Dysfunction during Oxidative Stress-induced Cell Death
Source: J Biol Chem. 2017 Feb 6;292(11):4727–42. doi: 10.1074/jbc.M116.759084 (PMC5377786; doi:10.1074/jbc.M116.759084)
Supplement: Supplemental Data [file supp_292_11_4727__index.html]

Glyceraldehyde-3-phosphate Dehydrogenase (GAPDH) Aggregation Causes Mitochondrial Dysfunction during Oxidative Stress-induced Cell Death — GAPDH Aggregation Mediates Mitochondrial Dysfunction — Supplemental Data 

# Glyceraldehyde-3-phosphate Dehydrogenase (GAPDH) Aggregation Causes Mitochondrial Dysfunction during Oxidative Stress-induced Cell Death

## Supplemental Data

- Supplemental Data
