# Supplementary material for: Glyceraldehyde-3-phosphate Dehydrogenase (GAPDH) Aggregation Causes Mitochondrial Dysfunction during Oxidative Stress-induced Cell Death
Source: J Biol Chem. 2017 Feb 6;292(11):4727–42. doi: 10.1074/jbc.M116.759084 (PMC5377786; doi:10.1074/jbc.M116.759084)

Supplementary Information

Glyceraldehyde-3-Phosphate Dehydrogenase (GAPDH) Aggregation Causes Mitochondrial Dysfunction During Oxidative Stress-Induced Cell Death

Hidemitsu Nakajima[1#]*, Masanori Itakura[1#], Takeya Kubo[1], Akihiro Kaneshige[1], Naoki Harada[2], Takeshi Izawa[3], Yasu-Taka Azuma[1], Mitsuru Kuwamura[3], Ryoichi Yamaji[2], and Tadayoshi Takeuchi[1]

From the [1]Laboratory of Veterinary Pharmacology, [2]Division of Applied Life Sciences, [3]Laboratory of Veterinary Pathology, Graduate School of Life and Environmental Science, Osaka Prefecture University, Izumisano, Osaka, Japan 5988531

[#]These authors contributed equally to this study.

***Corresponding author:** E-mail: hnakajima@vet.osakafu-u.ac.jp

SUPPLEMENTARY LEGENDS

Supplementary Figure S1. GAPDH aggregates do not act as mitochondrial trans-S-nitrosylases. *A*, GAPDH S-nitrosothiols were determined as described previously (1). [#] Treatment of soluble GAPDH with 1 mM S-nitroso-N-acetyl-$_{DL}$-penicillamine (SNAP) for 30 min was used as a positive control, according to the above-mentioned report. Data are presented as mean ± S.D (n = 4); **, P < 0.01, relative to soluble GAPDH, Dunnett's test. *B*, Mitochondrial trans-S-nitrosylation of GAPDH aggregates was assessed as previously described (2) with minor modifications. Isolated mitochondria were incubated with vehicle or GAPDH aggregates for 1 h before mitochondrial S-nitrosylated proteins were detected with a biotin-switch assay using avidin-HRP.

Supplementary Figure S2. NOC18-induced cell death is not regulated by nuclear translocation of GAPDH. *A*, Nuclear translocation of GAPDH in SH-SY5Y cells treated with NOC18 for 48 h as assessed by western blotting of nuclear fractions. The graph shows the values calculated as the nuclear GAPDH to H2B band intensity ratio. Data are presented as mean ± SD (n = 4); **, P < 0.01, relative to vehicle treatment, Student's t test. *B*, GAPDH (green) and DAPI (red) immunofluorescence. Merged signals are observed in NOC18-treated cells (right panel). Scale bar, 10 μm. *C*, Effect of doxycycline (DOX)-induced overexpression of WT- and C152A-GAPDH on nuclear translocation of endogenous GAPDH as assessed by western blotting of nuclear fractions. The total amounts of endogenous (Endo) and exogenous (Exo) nuclear GAPDH were increased by overexpression of WT-GAPDH, but not C152A-GAPDH. The graph presents the values calculated as the nuclear GAPDH to H2B band intensity ratio. Data are presented as mean ± SD (n = 4); **, P < 0.01, relative to DOX (-), Student's t test. *D*, GAPDH (green) and DAPI (red) immunofluorescence in SH-SY5Y cells treated with NOC18. Merged signals are augmented in WT-GAPDH-overexpressing cells. Scale bar, 10 μm. *E*, Effect of treatment with the GAPDH nuclear translocation inhibitor, deprenyl (Dep, 10 nM), on viability of WT- and C152A-GAPDH-overexpressing SH-SY5Y cells treated with NOC18 for 48 h. Data are presented as mean ± S.D (n = 4); **, P < 0.01, relative to DOX (-), Student's t test.

1. Ishii, T., Sunami, O., Nakajima, H., Nishio, H., Takeuchi, T., and Hata, F. (1999) Critical role of sulfenic acid formation of thiols in the inactivation of glyceraldehyde-3-phosphate dehydrogenase by nitric oxide. *Biochemical pharmacology* **58**, 133-143
2. Kohr, M. J., Murphy, E., and Steenbergen, C. (2014) Glyceraldehyde-3-phosphate dehydrogenase acts as a mitochondrial trans-S-nitrosylase in the heart. *PLoS One* **9**, e111448

Supplementary Figure S1

A

S-nitrosylation

	SNO/tetramer
Soluble GAPDH	0.10 ± 0.01
GAPDH aggregates	0.10 ± 0.02
Soluble GAPDH + SNAP#	3.98 ± 0.13**

B

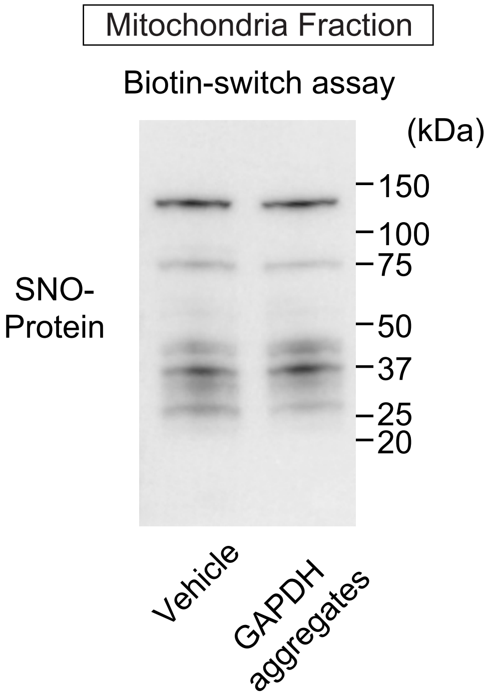

Supplementary Figure S2

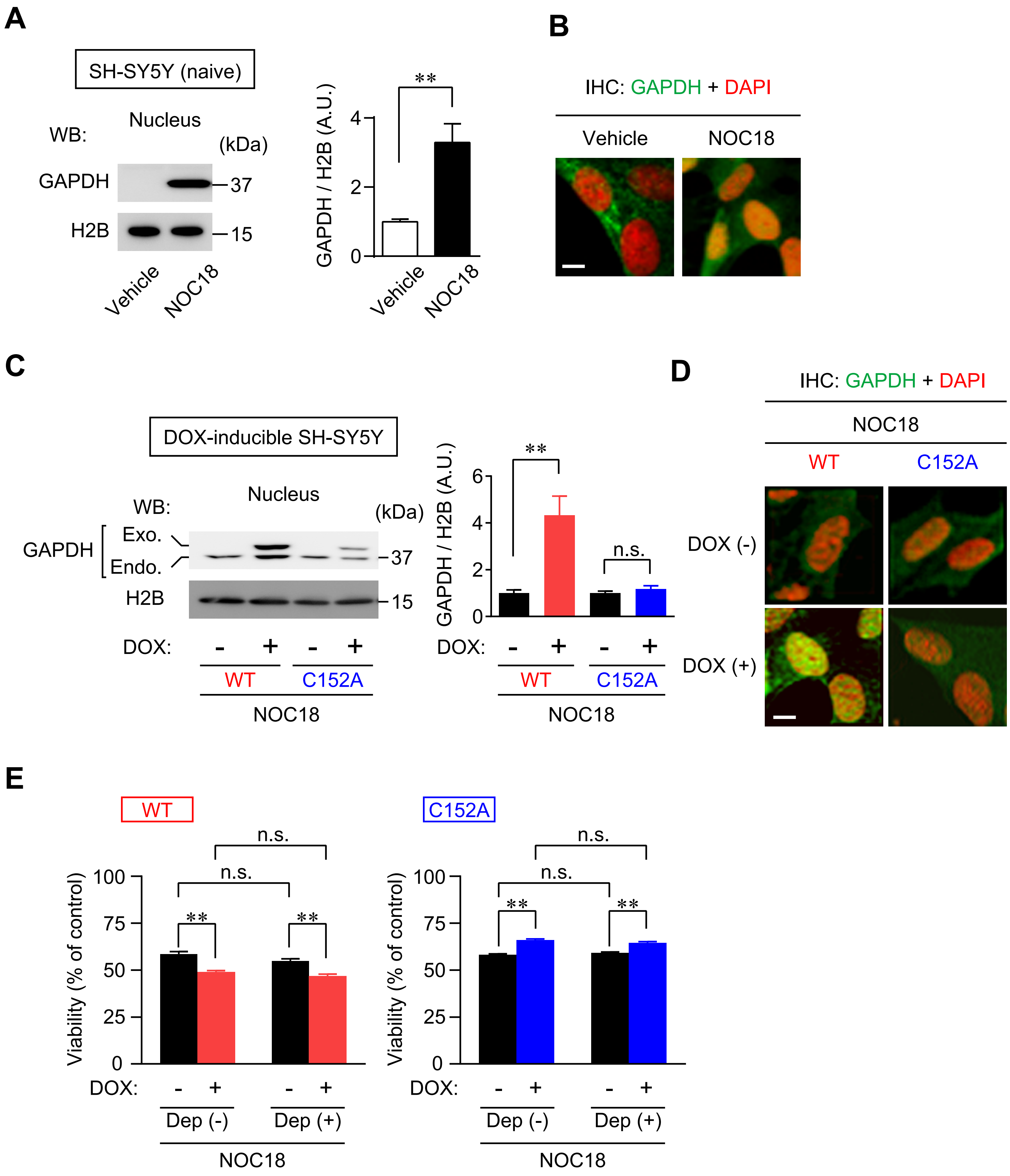

Supplement: Supplemental Data [file supp_M116.759084_suppdata.pdf]
